# Substitution of the premembrane and envelope protein genes of Modoc virus with the homologous sequences of West Nile virus generates a chimeric virus that replicates in vertebrate but not mosquito cells

**DOI:** 10.1186/1743-422X-11-150

**Published:** 2014-08-24

**Authors:** Rungrat Saiyasombat, Jimena Carrillo-Tripp, Wyatt Allen Miller, Peter J Bredenbeek, Bradley J Blitvich

**Affiliations:** Department of Veterinary Microbiology and Preventive Medicine, College of Veterinary Medicine, Iowa State University, Ames, Iowa USA; Department of Plant Pathology and Microbiology, College of Agriculture and Life Sciences, Iowa State University, Ames, Iowa USA; Department of Medical Microbiology, Leiden University Medical Center, Leiden, RC, NL-2300 The Netherlands

**Keywords:** Modoc virus, West Nile virus, Culex flavivirus, Chimeric flavivirus, Fusion PCR

## Abstract

**Background:**

Most known flaviviruses, including West Nile virus (WNV), are maintained in natural transmission cycles between hematophagous arthropods and vertebrate hosts. Other flaviviruses such as Modoc virus (MODV) and Culex flavivirus (CxFV) have host ranges restricted to vertebrates and insects, respectively. The genetic elements that modulate the differential host ranges and transmission cycles of these viruses have not been identified.

**Methods:**

Fusion polymerase chain reaction (PCR) was used to replace the capsid (C), premembrane (prM) and envelope (E) genes and the prM-E genes of a full-length MODV infectious cDNA clone with the corresponding regions of WNV and CxFV. Fusion products were directly transfected into baby hamster kidney-derived cells that stably express T7 RNA polymerase. At 4 days post-transfection, aliquots of each supernatant were inoculated onto vertebrate (BHK-21 and Vero) and mosquito (C6/36) cells which were then assayed for evidence of viral infection by reverse transcription-PCR, Western blot and plaque assay.

**Results:**

Chimeric virus was recovered in cells transfected with the fusion product containing the prM-E genes of WNV. The virus could infect vertebrate but not mosquito cells. The *in vitro* replication kinetics and yields of the chimeric virus were similar to MODV but the chimeric virus produced larger plaques. Chimeric virus was not recovered in cells transfected with any of the other fusion products.

**Conclusions:**

Our data indicate that genetic elements outside of the prM-E gene region of MODV condition its vertebrate-specific phenotype.

## Introduction

All viruses in the genus *Flavivirus* (family *Flaviviridae*) possess a single-stranded, positive-sense RNA genome of approximately 11 kb [1]. The genome contains a single open reading frame (ORF) flanked by 5’ and 3’ untranslated regions (UTRs) of ~100 and 400–700 nt, respectively [2]. The 5’ end of the genome contains a type I cap structure and the 3’ end is non-polyadenylated. The ORF encodes a single polyprotein that is co- and post-translationally cleaved to generate three structural proteins, designated the capsid (C), premembrane/membrane (prM/M) and envelope (E) proteins, and at least seven non-structural (NS) proteins in the gene order: 5′–C–prM(M)–E–NS1–NS2A–NS2B–NS3–NS4A–NS4B–NS5-3′ [1, 3]. Cleavage events are mediated by a combination of endoplasmic reticulum signalases, furin and the viral trypsin-like serine protease [1, 4, 5].

The flavivirus genome is packaged in an icosahedral nucleocapsid with multiple copies of the C protein [1]. The nucleocapsid is surrounded by a lipid envelope, acquired from the host cell, in which the prM(M) and E proteins are embedded. The E protein is required for receptor binding, host membrane fusion and viral assembly, while the prM protein protects the E protein from undergoing an irreversible conformational change as the virion is secreted through acidified sorting compartments [6–9]. RNA replication occurs in the cytoplasm in close association with the rough endoplasmic reticulum and requires the participation of several NS proteins including the viral helicase and protease (NS3), viral protease cofactor (NS2B) and RNA-dependent RNA polymerase and methyltransferase (NS5) [4, 10, 11].

Flaviviruses can be divided into three distinct groups based upon their mode of transmission [12, 13]. The first group is comprised of viruses that are transmitted horizontally between hematophagous arthropods and vertebrate hosts. This group can be further divided into mosquito-borne and tick-borne viruses. Examples of mosquito-borne flaviviruses include West Nile virus (WNV), dengue virus (DENV), yellow fever virus (YFV) and Japanese encephalitis virus (JEV), all of which are human pathogens of global concern [14]. Tick-borne flaviviruses associated with serious human disease include tick-borne encephalitis virus (TBEV), Langat virus (LGTV) and Powassan virus. Flaviviruses in the second group have no known arthropod vector (NKV) and are considered to be vertebrate-specific. NKV flaviviruses have been isolated exclusively from bats and rodents, and examples include Modoc virus (MODV) and Rio Bravo virus [15, 16]. The mechanism(s) by which NKV flaviviruses are maintained in nature is poorly defined but it has been suggested that they are transmitted between hosts by nasal and/or oral contact [17–19]. The final group is comprised of insect-specific flaviviruses (ISFs). These viruses are assumed to be insect-specific because they have been isolated from mosquitoes but do not replicate in mice or any vertebrate cell lines that have been tested. More than 20 ISFs have been discovered including Culex flavivirus (CxFV), cell fusing agent virus and Kamiti River virus [20–23]. Recent data indicate that ISFs are maintained in nature by transovarial transmission [24]. It is not known whether ISFs and NKV flaviviruses were originally arthropod-vertebrate flaviviruses that lost the ability to replicate in one host or if they are progenitor viruses from which the arthropod/vertebrate flaviviruses evolved, although the latter theory is favored [25, 26].

The evolutionary processes and underlying genetic basis for the differential host ranges and transmission cycles of flaviviruses have not been identified. Thus, the overall goal of this study is to characterize the *in vitro* host ranges of chimeric viruses constructed using representative viruses from the vertebrate-specific, insect-specific and arthropod/vertebrate flavivirus groups (MODV, CxFV and WNV, respectively) in order to increase our knowledge of the genetic elements that condition the vastly different host ranges and transmissibilities of these viruses.

## Materials and methods

### Cell lines

BSR-T7/5 cells, which are baby hamster kidney-derived cells that constitutively express T7 RNA polymerase [27], were kindly provided by Cathy Miller (Iowa State University). Baby hamster kidney (BHK-21), African Green Monkey kidney (Vero) and *Aedes albopictus* (C6/36) cells were obtained from the American Type Culture Collection (Manassas, VA). BSR-T7/5 and BHK-21 cells were cultured in minimum essential medium (Invitrogen, Carlsbad, CA), Vero cells were cultured in Dulbecco’s modified Eagle medium (Invitrogen) and C6/36 cells were cultured in Liebovitz L15 medium (Invitrogen). All media was supplemented with 10% fetal bovine serum (FBS), 2 mM L-glutamine, 100 units/ml penicillin and 100 μg/ml streptomycin. Mammalian cells were cultured at 37°C with 5% CO_2_ whereas C6/36 cells were cultured at 28°C.

### Viruses

pACNR-FLMODV, which contains full-length cDNA of MODV (strain M544) downstream of a T7 Ф2.5 promoter (Peter J. Bredenbeek, unpublished data), was used as template for fusion PCR reactions. The plasmid was also used to amplify the full-length product needed to generate MODV. WNV (strain NY99-flamingo382-99) was kindly provided by Aaron Brault (Centers for Disease Control and Prevention). CxFV (strain Iowa07) was originally isolated from *Culex pipiens* in Iowa in 2007 [28]. cDNAs were generated from WNV and CxFV RNA and used as template for fusion PCR reactions as described below.

### Construction of chimeric cDNAs

**Figure 1:**
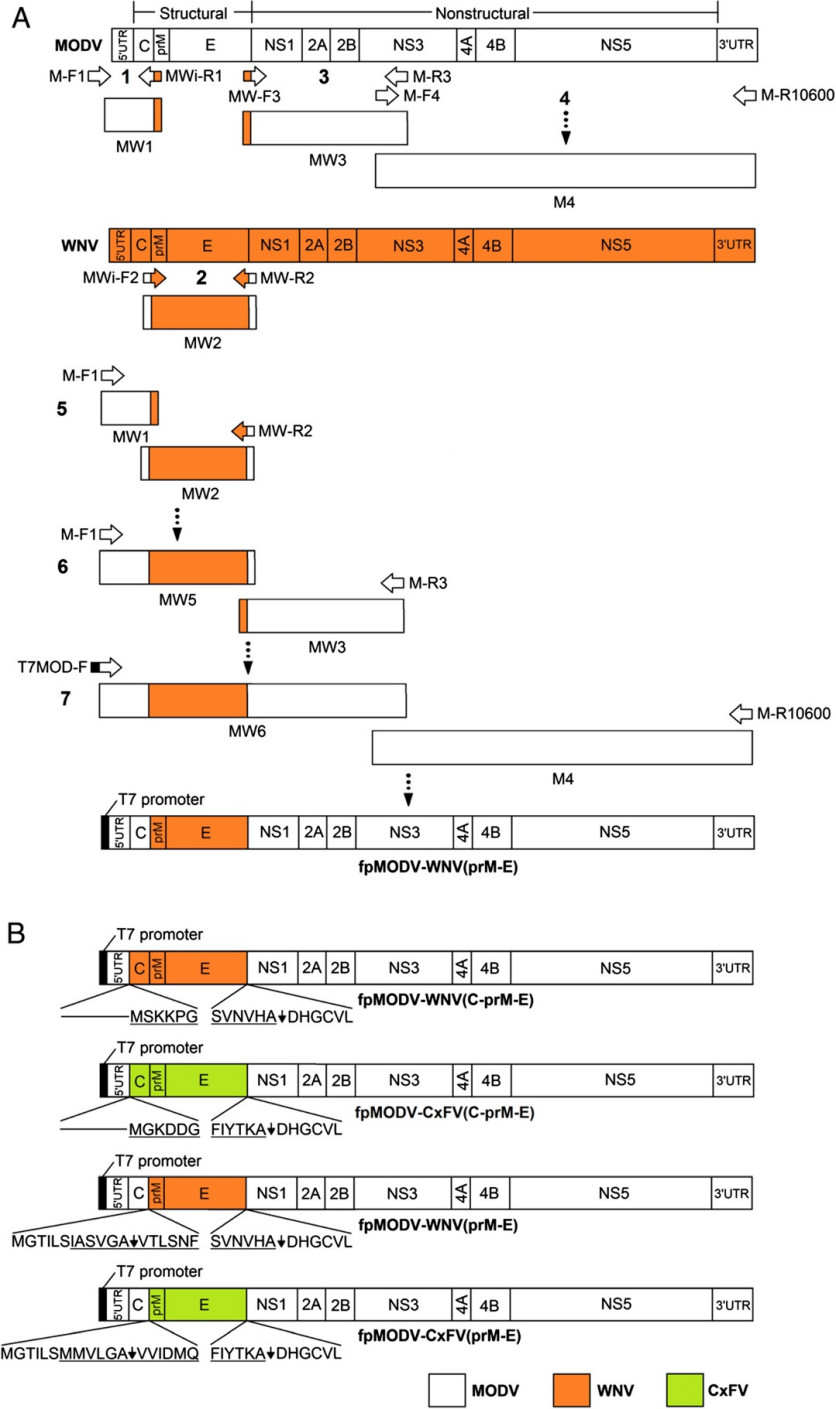
**Schematic of the fusion-PCR strategy used to generate viral chimeras. (A).** Strategy used to generate fpMODV-WNV(prM-E) in seven steps. The approximate location of primers and intermediate PCR products are shown on each viral genome (not scaled). Note that just viral sequences are depicted, the actual MODV template was pACNR-FLMODV while WNV template was viral cDNA (see materials and methods). All intermediate products and primers are further described in accompanying Table 1. Chimeric primers are represented by bicolor arrows. Steps 1–4: Products MW1, MW2, MW3 and M4 were generated by PCR with the indicated primers. These fragments were used as construction blocks in subsequent steps in fusion PCRs. Step 5: Products MW1 and MW2 were fused amplifying with primers M-F1 and MW-R2 to generate product MW5. Step 6: MW5 was fused with MW3 using primers M-F1 and M-R3 to give MW6. Step 7: In the final reaction, a full-length chimeric product was generated by fusing MW6 to M4 using primers T7-MOD-F and M-R10600. **(B).** Maps of final constructs highlighting the resulting amino acid chimeric sequences. Arrows indicate protease cleavage sites. Sequences from the heterologous viruses (WNV or CxFV) are underlined.

Four full-length chimeric flavivirus fusion products, designated fpMODV-WNV(C-prM-E), fpMODV-WNV(prM-E), fpMODV-CxFV(C-prM-E) and fpMODV-CxFV(prM-E), were generated by replacing the C-prM-E and prM-E genes of MODV with the homologous genes of WNV and CxFV. Four conventional PCRs and three fusion-PCRs were required to generate each full-length fusion product (Table 1). The process was facilitated by chimeric primers (half MODV sequence and half heterologous virus sequence) that worked as linkers to fuse the intermediate reaction products and subsequently assemble the final chimeras. The strategy used to construct fpMODV-WNV(prM-E) is depicted in Figure 1 and described below as an example of the chimeric viral cDNA construction process. In the first reaction, a 523 bp product (designated MW1) was amplified by PCR using pACNR-FLMODV as template, a forward primer (M-F1; see Tables 1 and 2) specific to the vector sequence upstream of the MODV 5’UTR and a chimeric reverse primer (MWi-R1) specific to the distal 3’ and 5’ ends of the MODV C and WNV prM genes, respectively. In the second reaction, a 2066 bp product (MW2) that contains the entire prM-E genes of WNV was amplified by RT-PCR using total RNA extracted from WNV-infected C6/36 cells as template, a forward chimeric primer (MWi-F2) specific to the sequences at the distal 3’ and 5’ ends of the MODV C and WNV prM genes, respectively and a reverse chimeric primer (MW-R2) specific to the sequences at the distal 3’ and 5’ ends of the WNV E and MODV NS1 genes, respectively. In the third reaction, a 2575 bp product (MW3) that contains the entire NS1-NS2A-NS2B genes and part of the NS3 gene of MODV was amplified by PCR using pACNR-FLMODV as template, a forward chimeric primer (MW-F3) specific to the WNV E and MODV NS1 genes and a reverse primer (M-R3) specific to an internal region of the MODV NS3 gene. In reaction four, the remainder of the NS3 gene and the entire NS4A-NS4B-NS5-3’UTR region of MODV was amplified by PCR using pACNR-FLMODV as template and MODV-specific forward and reverse primers (M-F4 and M-R10600, respectively) to give a 6227 bp product (M4). Reaction 5 was a fusion-PCR in which MW1 and MW2 were used as templates and M-F1 and MW-R2 as primers for the generation of a 2542 bp product designated MW5. Reaction 6 was another fusion-PCR in which MW3 and MW5 were used as templates and M-F1 and M-R3 as primers for the generation of a 5079 bp product designated MW6. In the final reaction, a full-length 10,708 bp chimeric fusion product designated fpMODV-WNV(prM-E) was generated by fusion-PCR using M4 and MW6 as templates and T7-MOD-F and M-R10600 as forward and reverse primers, respectively. The 5’ end of T7-MOD-F contains the T7 promoter sequence. A similar strategy was adopted for the construction of fpMODV-WNV(C-prM-E), fpMODV-CxFV(C-prM-E) and fpMODV-CxFV(prM-E) with the primers used in these experiments and the sizes of the resulting amplification products denoted in Tables 1 and 2. Full-length MODV was also amplified in a single PCR using pACNR-FLMODV as template, T7-MOD-F as the forward primer and M-R10600 as the reverse primer (Table 2). All full-length products were purified by phenol/chloroform extraction and ethanol precipitation, and sequenced across the junctions using overlapping primers for junction verification.

**Table 1:** **PCR products generated during the construction of full-length flavivirus chimeric DNAs**

Reaction no.	Reaction type	Primers (forward, reverse)	PCR product	
			Name	Size (bp)
1a	PCR	M-F1, MW-R1	MW1’	191
1b		M-F1, MC-R1	MC1’	194
1c		M-F1, MWi-R1	MW1	523
1d		M-F1, MCi-R1	MC1	521
2a	RT-PCR	MW-F2, MW-R2	MW2’	2,415
2b		MC-F2, MC-R2	MC2’	2,167
2c		MWi-F2, MWR2	MW2	2,066
2d		MCi-F2, MCR2	MC2	1,777
3a,c	PCR	MW-F3, M-R3	MW3	2,575
3b,d		MC-F3, M-R3	MC3	2,580
4a-d	PCR	M-F4, M-R10600	M4	6,227
5a	Fusion-PCR	M-F1, MW-R2	MW5’	2,563
5b		M-F1, MC-R2	MC5’	2,320
5c		M-F1, MW-R2	MW5	2,542
5d		M-F1, MC-R2	MC5	2,251
6a	Fusion-PCR	M-F1, M-R3	MW6’	5,100
6b		M-F1, M-R3	MC6’	4,854
6c		M-F1, M-R3	MW6	5,079
6d		M-F1, M-R3	MC6	4,785
7a	Fusion-PCR	T7MOD-F, M-R10600	fpMODV-WNV(C-prM-E)	10,730
7b		T7MOD-F, M-R10600	fpMODV-WNV(prM-E)	10,708
7c		T7MOD-F, M-R10600	fpMODV-CxFV(C-prM-E)	10,484
7d		T7MOD-F, M-R10600	fpMODV-CxFV(prM-E)	10,415

Reactions ending with a, b, c and d were used to generate fpMODV-WNV(C-prM-E), fpMODV-CxFV(C-prM-E), fpMODV-WNV(prM-E) and fpMODV-CxFV(prM-E), respectively.

**Table 2:** **Primers used during the construction of full-length flavivirus chimeric DNAs**

Primer	Polarity	Sequence ^a^	Target
M-F1	Sense	5’ACATTTCCCCGAAAAGTGCCACCTGACGTCTCGAC3’	Cloning vector
MW-R1	Antisense	5’*CCTCCTGGTTTCTTAGACAT*TCCCGCCACAAAAAGTGG3’	*WNV*/MODV
MWi-R1	Antisense	5’*TAACTGCTCCTACGCTGGCGAT*TGACAATATGGTTCCCATCATCC3’	*WNV*/MODV
MC-R1	Antisense	5’*CTTACCGTCGTCCTTTCCCAT*TCCCGCCACAAAAAGTGG3’	*CxFV*/MODV
MCi-R1	Antisense	5’*ACGGCGCCCAGCACCATCAT*TGACAATATGGTTCCCATCATC3’	*CxFV*/MODV
MW-F2	Sense	5’CCACTTTTTGTGGCGGGA*ATGTCTAAGAAACCAGGAGG*3’	MODV/*WNV*
MWi-F2	Sense	5’ATGGATGATGGGAACCATATTGTCA*ATCGCCAGCGTAGGAGCAG*3’	MODV/*WNV*
MC-F2	Sense	5’CCACTTTTTGTGGCGGGA*ATGGGAAAGGACGACGGTAAG*3’	MODV/*CxFV*
MCi-F2	Sense	5’ATATGGATGATGGGAACCATATTGTCA*ATGATGGTGCTGGGCGCCGTC*3’	MODV/*CxFV*
MW-R2	Antisense	5’CAAGGACACAGCCATGATC*AGCGTGCACGTTCACGGAG*3’	MODV/*WNV*
MC-R2	Antisense	5’CATCAAGGACACAGCCATGATC*TGCCTTGGTGTAGATAAAGTATCC*3’	MODV/*CxFV*
MW-F3	Sense	5’*CTCCGTGAACGTGCACGCT*GATCATGGCTGTGTCCTTG3’	*WNV*/MODV
MC-F3	Sense	5’*GGATACTTTATCTACACCAAGGCA*GATCATGGCTGTGTCCTTGATG3’	*CxFV*/MODV
M-R3	Antisense	5’TCCATTTGCATTGATGACTGGAGAACCAGATGAACCAGGAGG3’	MODV
M-F4	Sense	5’AGACTCTTATTCTTGGGGTGGG3’	MODV
T7MOD-F	Sense	5’**TAATACGACTCACTATAGG**AGTTGATCCTGCCAGCGGTG3’	**T7**/MODV
M-R10600	Antisense	5’AGCGGAGGTCATATTCATGACCACACAGATTACATG3’	MODV

^a^Heterologous virus sequences are italicized in chimeric primers, T7 promoter sequence is bolded.

### Transfections and virus recovery

Full-length PCR products (chimeras and full-length MODV) were transfected directly into BSR-T7/5 cells (which stably express T7 RNA polymerase) in order to avoid the *in vitro* transcription step. BSR-T7/5 cells were seeded into 60 mm^2^ sterile plates and incubated until there were approximately 9.5x10^5^ cells per plate. Cells were transfected with 5 μg of purified full-length flavivirus cDNA mixed with 500 μl of serum-free Opti-MEM (Invitrogen) and 15 μl of TransIT-LT1 transfection reagent (Mirus Bio, Wisconsin) according to the manufacturer’s instructions. For those constructs that failed to generate virus, at least three independent transfections were performed and the full-length MODV construct was included as a positive control in each experiment. Transfected BSR-T7/5 cells were incubated for 4 days then aliquots of each supernatant were collected and inoculated onto subconfluent monolayers of Vero, BHK-21 and C6/36 cells. Several more passages were performed in the same cell type or, where specified, an alternate cell type. Cells were monitored daily for cytopathic effect (CPE). Cell monolayers and supernatants were harvested when 50-70% of the cells exhibited CPE. If CPE was not observed, cells were harvested at 7 to 9 days post-inoculation (p.i.), with the exception of BHK-21 cells which were harvested at 4 days p.i. since all BHK-21 cell cultures (including the negative control cultures) displayed considerable cell death at this time.

### Reverse transcription-polymerase chain reaction

Total RNA was extracted from cell monolayers and supernatants using Trizol Reagent (Invitrogen) and the QIAamp viral RNA mini kit (Qiagen, Valencia, CA), respectively. Complementary DNAs were generated using Superscript III reverse transcriptase (Invitrogen). Where specified, RNA templates were treated with deoxyribonuclease I (DNase I; Invitrogen) prior to reverse transcriptions. PCRs were performed using high fidelity *Taq* polymerase (Invitrogen). MODV, WNV and CxFV-specific primers were designed using published sequences (Genbank Accession No. AJ242984, AF196835 and FJ663034, respectively). PCR products were examined by 0.8-1% agarose gel electrophoresis, purified using QIAquick spin columns (Qiagen) and sequenced using a 3730x1 DNA sequencer (Applied Biosystems, Foster City, CA).

### Preparation of protein lysates

BHK-21, Vero and C6/36 cell monolayers, approaching confluency in 75 cm^2^ flasks, were inoculated with parental or chimeric virus at a multiplicity of infection (m.o.i) of 0.1 and incubated for 4 days (BHK-21 cells) or 7 days (Vero and C6/36 cells). Cells were scraped from the surface of the flask, clarified by centrifugation (10,000 g, 10 min, 4°C), washed twice with cold phosphate-buffered saline (PBS), resuspended in lysing buffer [10 mM Tris–HCl pH 7.5, 150 mM NaC1, 5 mM EDTA, 1% sodium deoxycholate, 1% Triton X-100, 0.1% SDS and a cocktail of protease inhibitors (Sigma, St. Louis, MO)] and placed on ice for 15 min. Samples were microfuged at 4°C for 15 min and supernatants collected and stored at -80°C.

### Western blots

Protein samples were mixed with an equal volume of reducing sample buffer, heated (95°C for 5 min) and resolved on 8-16% Tris-glycine gels (Invitrogen). Proteins were transferred to 0.45 μm nitrocellulose membranes (Invitrogen) following published protocols [29]. Membranes were blocked overnight at 4°C in phosphate-buffered saline (PBS, pH 7.2) with 5% (wt/vol) non-fat dried milk. Membranes were incubated with (i) 1/100 immune ascitic fluid obtained from mice inoculated with MODV (American Type Culture Collection) or a (ii) 1/100 pooled suspension of anti-WNV E protein monoclonal antibodies 3.67G and 3.91D (Millipore, Billerica, MA) for 1 hr at room temperature. Membranes were then washed and incubated with 1/2000 horseradish peroxidase-conjugated anti-mouse IgG antibody (Invitrogen) for 1 hr at room temperature. Specifically bound antibody was visualized using 3,3’-diaminobenzidine (0.05% in PBS with 0.018% H_2_O_2_).

### Plaque assays

Viruses were subjected to serial tenfold dilutions, inoculated onto confluent monolayers of Vero cells in 35-mm culture dishes then incubated at 37°C for 60 min. Three milliliters of neutral red-deficient minimum essential medium (Invitrogen) supplemented with 2% FBS, antibiotics and 1% agar were added to each well. Plates were incubated at 37°C for 3, 5 or 7 days for WNV, chimeric virus and MODV plaque assays, respectively. Another 3 ml of the same medium containing 0.22% neutral red was then added to each well, and plaques were counted 24 h later. Viral titers were expressed as plaque-forming units per milliliter (pfu/ml).

### Plaque morphology comparisons

Viruses were inoculated onto confluent monolayers of Vero cells in 35-mm culture dishes then incubated at 37°C for 60 min. Three milliliters of neutral red-deficient minimum essential medium (Invitrogen) supplemented with 2% FBS, antibiotics and 1% agar were added to each well, and plates were incubated at 37°C for 3, 5 or 7 days. To fix the cells, 2 ml of 10% formaldehyde was added directly onto each agar overlay and the plates were incubated at 37°C for 60 min. Agar overlays were gently removed, and 0.5 ml of 0.25% crystal violet (w/v) in 20% methanol was added to each well. Once the desired intensity was reached, plates were rinsed several times with tap water and photographed.

### Growth curve comparisons

Subconfluent monolayers of Vero cells in 150 cm^2^ flasks were inoculated with chimeric virus, MODV or WNV at a m.o.i. of 0.1 Supernatants were collected daily for 7 days, clarified by centrifugation (10,000 g, 10 min, 4°C) and stored in aliquots at -80°C until titrated by plaque assay. Three independent experiments were performed. Within each experiment, six replicates of each virus/dilution/timepoint were tested. Data were used to calculate mean viral titers ± 1 standard deviation.

## Results

We initially attempted to create chimeric viruses by replacing the C-prM-E and prM-E genes of the MODV infectious cDNA clone with the corresponding sequences of WNV and CxFV using restriction enzyme digestion and direct cloning strategies (data not shown). More than 2,000 bacterial colonies were screened by PCR but none contained full-length C-prM-E or prM-E sequences from the heterologous virus. Approximately 10% of the colonies contained WNV or CxFV sequences that had been truncated or contained transposon insertions. These findings led us to speculate that the structural genes of WNV and CxFV are toxic to *E. coli* cells. In order to overcome this problem, the use of bacteria and traditional cloning was replaced by a fusion PCR-based strategy coupled to an *in vitro* transcription-free system for virus production. Similar methodologies have been developed for other arboviruses [30, 31].

**Figure 2:**
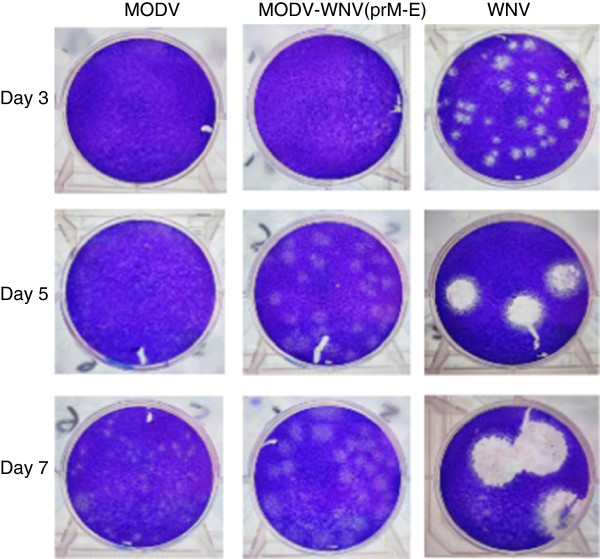
**Comparison of the plaque morphologies of MODV-WNV(prM-E) and the parental viruses in Vero cells.** Confluent monolayers of Vero cells in six-well plates were inoculated with MODV-WNV(prM-E), MODV or WNV. Cells were fixed and plaques were visualized by staining with crystal violet at 3, 5 and 7 days p.i. Images were transferred into Microsoft Photoshop and plaque diameters were measured. The chimeric virus had been passaged one in BHK-21 cells and twice in Vero cells prior to this experiment.

**Figure 3:**
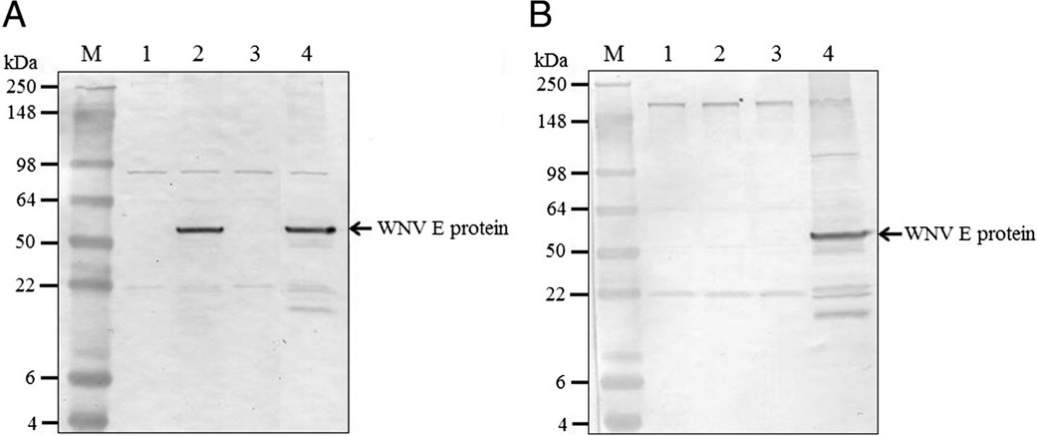
**Western blot analysis reveals the presence of WNV antigen in Vero cells, but not C6/36 cells, inoculated with MODV-WNV(prM-E).** Lysates were prepared from **(A)** Vero and **(B)** C6/36 cells that had been mock-inoculated (lane 1) or inoculated with chimeric virus (lane 2), MODV (lane 3) or WNV (lane 4) at a m.o.i. of 0.1. Lysates were harvested at 7 days p.i. and equal amounts of protein were resolved on 8-16% Tris-glycine gels and immunoblotted using a pooled suspension of anti-WNV E protein monoclonal antibodies. M denotes the SDS PAGE low-range molecular weight standards (Invitrogen). The arrow shows the expected migration position of the WNV E protein (molecular weight: 53 KDa).

**Figure 4:**
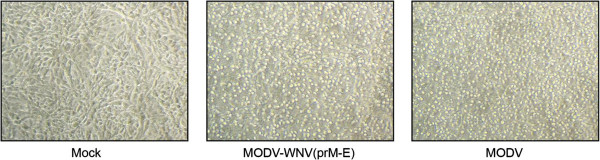
**Detection of cytopathic effect in Vero cells inoculated with MODV-WNV(prM-E).** MODV-WNV(prM-E) and MODV that had been passaged twice in Vero cells were inoculated onto fresh monolayers of Vero cells that were monitored for 5 or 8 days, respectively. Mock-infected Vero cells that were incubated for 5 days were also included. Magnification = 100×.

Four full-length chimeric flavivirus fusion products, designated fpMODV-WNV(C-prM-E), fpMODV-WNV(prM-E), fpMODV-CxFV(C-prM-E) and fpMODV-CxFV(prM-E), were generated by substituting the C-prM-E and prM-E genes of MODV with the corresponding regions of WNV and CxFV. The strategy used to generate fpMODV-WNV(prM-E) is shown in Figure 1, and a similar approach was used to create the three other full-length fusion products. Full-length constructs were transfected into BSR-T7/5 cells. Since all of the full-length products contain a T7 promoter at the 5’ end and because BSR-T7/5 cells constitutively express T7 RNA polymerase [27], there was no need to perform an *in vitro* transcription before the transfection. At 4 days post-transfection, aliquots of each supernatant were collected and inoculated onto Vero, BHK-21 and C6/36 cells. Supernatants were harvested from these cell cultures at 4 days p.i. (BHK-21 cells) or 7 to 9 days p.i. (Vero and C6/36 cells) then passed several more times in the same cell type (or, where specified, a different cell type). Cell monolayers and supernatants were harvested and tested for evidence of virus infection by RT-PCR, Western blot and plaque assay.Chimeric virus was successfully generated in BSR-T7/5 cells transfected with fpMODV-WNV(prM-E). None of the other full-length chimeric flavivirus fusion products produced detectable virus under these conditions. The chimeric virus, designated MODV-WNV(prM-E), possessed the capacity to infect and replicate within vertebrate but not mosquito cells (Figures 2 and 3). Supernatants harvested from MODV-WNV(prM-E)-infected Vero and BHK-21 cells produced distinct plaques in Vero cells (Figure 2) whereas supernatants harvested from C6/36 cells inoculated with the chimeric virus did not (data not shown). MODV-WNV(prM-E) plaques were larger and could be visualized earlier than MODV plaques but were smaller and visualized later than WNV plaques. At 3 days p.i., MODV and MODV-WNV(prM-E) plaques were barely visible (and too small to be measured accurately) whereas WNV plaques had a mean diameter ± 1 standard deviation of 1.9 ± 0.15 mm. At 5 days p.i., MODV, MODV-WNV(prM-E) and WNV plaques were 0.1 ± 0.02, 1.8 ± 0.14 and 7.5 ± 0.46 mm in diameter, respectively. At 7 days p.i., MODV, MODV-WNV(prM-E) and WNV plaques were 0.9 ± 0.11, 2.9 ± 0.20 and 11.7 ± 0.85 mm in diameter, respectively. Analyses of variance (ANOVA) F-test showed significant difference among the plaque sizes of the three viruses on both day 5 (F = 5833.24, DF = 2, 87, p-value <0.0001) and day 7 (F = 3705.42, DF = 2, 67, p-value <0.0001). Post-hoc Tukey’s t-test showed that all pairwise comparisons were significant (adjusted p-value <0.0001).Chimeric flavivirus RNA was detected by RT-PCR in supernatants harvested from Vero and BHK-21 cells, but not C6/36 cells, that had been inoculated with MODV-WNV(prM-E) (data not shown). Nucleotide sequencing of the RT-PCR products confirmed these findings. WNV antigen was detected in cell lysates harvested from MODV-WNV(prM-E)-inoculated Vero cells, but not C6/36 cells, in Western blots performed using WNV-specific monoclonal antibodies (Figure 3). MODV antigen was not detected by Western blot in any cells inoculated with chimeric virus or MODV (both fusion-PCR-derived and wild-type MODV) when commercial immune ascitic fluid obtained from mice infected with MODV was used, possibly because the mice failed to generate a sufficient immune response.It is interesting to note that the chimeric virus did not always produce CPE in Vero cells. CPE was not observed in Vero cells directly inoculated with supernatants harvested from fpMODV-WNV(prM-E)-transfected BSR-T7 cells. An additional passage in Vero cells also failed to result in CPE despite the detection of chimeric viral RNA in these cultures by RT-PCR. However, after a third passage in Vero cells, CPE was clearly observed. In contrast, CPE was observed after one passage in Vero cells when the chimeric virus first underwent one passage in BHK-21 cells (Figure 4).

We sequenced the complete C-prM-E genes of chimeric virus before and after it had been subjected to multiple cell culture passages to assess the genetic stability of the virus as well as to determine whether the acquisition of mutations within the structural genes could explain why some virus stocks possessed the ability to cause CPE in Vero cells while others did not. First, the entire C-prM-E region of MODV-WNV(prM-E) harvested from transfected BSR-T7/5 cell cultures were sequenced, and shown to contain one transition (coordinate 1457) resulting in a conservative substitution when compared to the corresponding region of the parental WNV (Table 3). We also sequenced the C-prM-E genes of chimeric virus that had undergone one passage in BHK-21 cells followed by two passages in Vero cells. Three additional transitions were identified; two mutations were silent and the other was conservative. In addition, we sequenced the C-prM-E region of chimeric virus that had undergone three passages in Vero cells and identified the change in nucleotide coordinate 1457 and four extra substitutions. One mutation was silent, one conservative and two were non-conservative.

**Table 3:** **Mutations accrued in the C-prM-E genes of MODV-WNV(prM-E) during transfection and passage in designated cell types**

Passage history	Nucleotide position	Amino acid position	Nucleotide change	Amino acid change
Original Inoculum (BSR-T7)	1457	E-167	C → T	Leu → Phe
BHK-21 + Vero + Vero	323	C-72	T → C	Silent
	1457	E-167	C → T	Leu → Phe
	1771	E-271	T → C	Silent
	2372	E-472	A → G	Met → Val
Vero + Vero + Vero	462	prM-2	C → T	Thr → Ile
	1307	E-117	G → A	Ala → Thr
	1457	E-167	C → T	Leu → Phe
	1894	E-216	T → C	Silent
	2261	E-435	T → C	Phe → Leu

**Figure 5:**
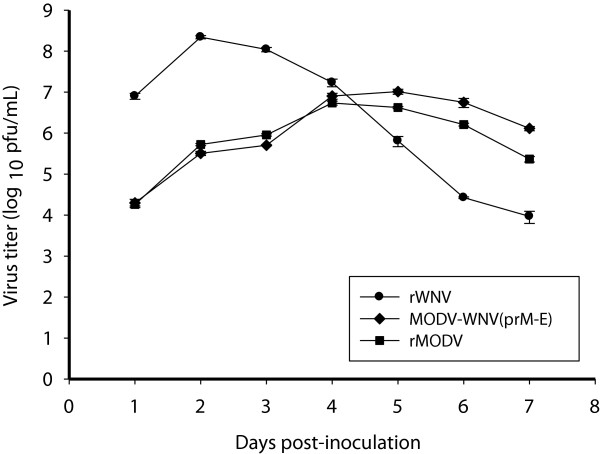
**Comparison of the replication kinetics of MODV-WNV(prM-E), MODV and WNV in Vero cells.** Subconfluent monolayers of Vero cells were inoculated with MODV-WNV(prM-E), MODV and WNV at a m.o.i of 0.1. Supernatants were collected daily for 7 days and tested by plaque assay. MODV-WNV(prM-E) had been passaged once in BHK-21 cells and once in Vero cells prior to the experiments.

MODV-WNV(prM-E) and MODV demonstrated similar replication kinetics and yields in Vero cells while WNV replicated faster and produced a higher peak titer (Figure 5). The chimeric virus and MODV reached mean peak titers of 7 (±0.06) log_10_ pfu/ml at 5 days p.i. and 6.7 (±0.05) log_10_ pfu/ml at 4 days p.i., respectively. In contrast, the mean peak titer for WNV was 22 to 48-fold higher and occurred 2 to 3 days earlier.

## Discussion

Most chimeric flaviviruses have been developed for vaccine purposes. In these studies, live-attenuated vaccine candidates were created by inserting specific genetic elements (typically the prM-E genes) of the flavivirus of interest into a full-length infectious cDNA backbone of another flavivirus such as the YFV vaccine vector, YFV-17D, or an attenuated strain of DENV [32–38]. The construction and characterization of chimeric flaviviruses has also provided critical information on the genetic elements that modulate the differential vector ranges, transmissibilities and disease phenotypes of divergent flaviviruses. Several of these studies have been performed using representative flaviviruses from the tick-borne and mosquito-borne groups [39–44]. More pertinent to this investigation are the few studies that describe the construction and characterization of viral chimeras between NKV and arthropod/vertebrate flaviviruses [45–47]. Five chimeric flaviviruses have now been created between viruses from these two groups. The first chimeric virus was generated by substituting the prM-E genes of an infectious YFV cDNA infectious clone with the homologous genes of MODV [46] and the second contains the prM-E genes of MODV in a DENV-2 backbone [45]. Both chimeric viruses replicated in C6/36 cells indicating that the inability of NKV flaviviruses to infect mosquito cells is not mediated by the viral envelope but by a post-entry event. Two more chimeric viruses were constructed by replacing the conserved pentanucleotide sequence (CPS) or variable region (VR) of the 3’ UTR of a DENV-4 infectious clone with the corresponding region of MODV. Both viruses could infect C6/36 cells and adult mosquitoes at similar efficiencies to DENV-4 suggesting that the CPS and VR of mosquito/vertebrate flaviviruses are not required for mosquito infectivity. We too have successfully created a chimeric virus using a NKV and mosquito-borne flavivirus but, unlike the above studies, our virus was constructed using the vertebrate-specific virus as the backbone. The virus, designated MODV-WNV(prM-E), was created by replacing the prM-E genes of a MODV infectious clone with the corresponding sequences of WNV. MODV-WNV(prM-E) possesses the capacity to infect and replicate within vertebrate but not mosquito cell cultures indicating that there are sequence elements outside of the prM-E region that dictate the vertebrate-specific host range of MODV. However, it is important to note that the mosquito cells were cultured at a much lower temperature than the mammalian cells and thus, it is not known whether MODV-WNV(prM-E) was unable to infect mosquito cells due to a cell tropism restriction or a temperature-dependent restriction.

The fusion product designated fpMODV-CxFV(prM-E), which was created by replacing the prM and E genes of MODV with the homologous sequences of CxFV, failed to yield detectable virus. This finding is in contrast to the numerous studies that report the successful production of chimeric virus after the prM-E genes of one flavivirus are replaced with those of another [42, 48–54]. However, all of these studies were performed with flaviviruses that possess at least one common host. Indeed, although chimeric viruses have been created between viruses as divergent as tick- and mosquito-borne flaviviruses, and NKV and mosquito-borne flaviviruses, all viruses within these groups possess the ability to replicate within vertebrate cells. In contrast, ISFs and NKV flaviviruses do not possess a common host by virtue of their insect and vertebrate-specific phenotypes. Thus, the generation of chimeric viruses between ISFs and NKV flaviviruses may not be achievable or, at the very least, will prove extremely challenging because their genomes and resulting translation products may be fundamentally incompatible as a consequence of their evolutionary divergence and specialization to vastly different hosts.

Conserved complementary cyclization sequences reside within the capsid gene and 3’ UTR of the flavivirus genome. These sequences interact with one another to facilitate genome cyclization and are essential for viral replication [55, 56]. Thus, one explanation for the inability to produce infectious virus with the fusion products containing the C-prM-E genes of WNV and CxFV is because the genome cyclization elements within the 3’ UTR of MODV and the C gene of the alternate virus do not have sufficient complementary to support genome cyclization. In this regard, replacement of the 3’UTR of a DENV-4 infectious clone with the corresponding region of MODV also failed to produce virus [47]. Virus was also unable to be recovered when both UTRs as well as the C gene of DENV-4 were replaced with the corresponding regions of either LGTV or MODV, despite the presence of complementary cyclization sequences [44, 47]. The authors speculated that infectious virus was not produced because fundamental incompatibilities exist between the UTRs and replication complexes of highly divergent (e.g. mosquito-borne, tick-borne and vertebrate-specific) flaviviruses. However, C-prM-E gene substitutions between divergent flaviviruses have occasionally proven successful; Pletnev and colleagues produced chimeric virus after replacing all three structural genes of DENV-4 with those of TBEV [41].

The inability to produce chimeric virus with fpMODV-CxFV(prM-E), fpMODV-CxFV(C-prM-E) and fpMODV-WNV(C-prM-E) is unlikely due to aberrant replication complex formation. Assembly of the viral replication complex should not have been impeded due to mismatches between the various viral and cellular proteins that interact during this process because no nonstructural gene substitutions were made. It is also unlikely that correct proteolytic processing of the chimeric polyproteins could not occur. Amino acid sequence alignments have shown that the predicted cleavage sites required for proteolytic cleavage of the CxFV and MODV polyproteins are similar to one another and to those of WNV and other dual-host flaviviruses [22, 57–59]. Although the junctions of all four constructs were sequenced and shown to contain no nucleotide errors, these constructs were not sequenced in their entirety and thus, we cannot dismiss the possibility that the non-viable constructs contained lethal mutations outside the junctions that occurred during one of the PCR amplifications. Another explanation why fpMODV-CxFV(C-prM-E) and fpMODV-WNV(C-prM-E) failed to produce virus is because the encapsidation signal sequence of MODV (which, as with all flaviviruses, remains to be identified [60]) is not recognized by the capsid proteins of WNV or CxFV.

The replication kinetics and yields of MODV-WNV(prM-E) in Vero cells were similar to those of MODV. These data suggest that genetic elements outside of the prM-E region dictate the *in vitro* replication profiles of NKV flaviviruses in vertebrate cells. Other studies have also shown that chimeric flaviviruses generated by prM-E gene substitutions exhibit replication kinetics and yields similar to the virus from which the nonstructural genes were derived but distinct from the virus that contributed the prM-E sequences [46, 51, 61]. For instance, the *in vitro* replication kinetics of a chimeric virus that possessed the prM-E genes of MODV in a YFV-17D backbone were similar to those of YFV-17D but distinct from MODV which reached a higher peak titer [46]. Although the chimeric virus and MODV displayed similar *in vitro* replication kinetics, these two viruses exhibited differential plaque morphologies in Vero cells. MODV-WNV(prM-E) plaques were at least threefold larger than MODV plaques but approximately fourfold smaller than WNV plaques. These findings indicate that genetic elements both within and outside of the prM-E region modulate the plaque sizes of NKV flaviviruses. These findings differ from most other studies which compare the plaque sizes of chimeric flaviviruses generated by prM-E gene substitutions to those of both parental viruses. Usually prM-E gene substitutions generate chimeric viruses that produce plaques that are indistinguishable from one of the parental viruses [51, 62–64] or are smaller than both parental viruses [46, 65, 66]. However, replacement of the prM-E genes of JEV with those of DENV-4 produced a chimeric virus which, like our chimeric virus, exhibited an intermediate plaque phenotype; the chimeric virus produced plaques that were smaller than JEV but larger than DENV-4 in mammalian cells [67].

MODV-WNV(prM-E) did not always cause CPE in Vero cells, and the occurrence of CPE appeared dependent on the passage history of the virus. MODV-WNV(prM-E) was able to induce CPE after a single passage in Vero cells if it had first been cultured in BHK-21 cells. In contrast, CPE did not occur in Vero cells until the third passage when the virus had not been passaged in BHK-21 cells. One explanation for these findings is that MODV-WNV(prM-E) replicates more efficiently in BHK-21 cells as compared to Vero cells, possibly because it is a rodent cell line and most of the chimeric flaviviral genome was acquired from a virus with a natural host range that is apparently restricted to rodents. Alternatively, repeated passaging of the virus in Vero cells could have resulted in the accumulation of mutations that altered its ability to induce CPE in this cell type. In this regard, the C-prM-E gene sequence of chimeric virus derived from the original inoculum contained one non-synonymous mutation when compared to the corresponding regions of parental viruses while chimeric viruses that had undergone three passages in BHK-21 and/or Vero cells acquired three to four additional mutations in the structural gene region. Whether these mutations, or mutations that may have occurred elsewhere in the viral genome, altered the ability of the virus to induce CPE is not known but it does offer a likely explanation.

In summary, we report the first chimeric flavivirus to be constructed using a NKV flavivirus as the backbone. We also report the first attempts to create a chimeric flavivirus between an ISF and NKV flavivirus. Two constructs were generated, including one that contains the CxFV prM-E genes in a MODV backbone, but neither yielded detectable virus. Most success in the generation of chimeric flaviviruses has been achieved through prM-E gene substitutions. However, unlike our study, all previous studies were performed using flaviviruses that share a common host. These findings indicate that the successful generation of chimeric viruses between ISFs and NKV flaviviruses will prove extremely challenging due to the evolutionary divergence and differential host ranges of these viruses.
